# Mechanism of Action and Initial, *In Vitro* SAR of an Inhibitor of the *Shigella flexneri* Virulence Regulator VirF

**DOI:** 10.1371/journal.pone.0137410

**Published:** 2015-09-09

**Authors:** Anthony A. Emanuele, George A. Garcia

**Affiliations:** Department of Medicinal Chemistry, College of Pharmacy, University of Michigan, Ann Arbor, MI, United States of America; New York State Dept. Health, UNITED STATES

## Abstract

*Shigella* spp. are among the main causative agents of acute diarrheal illness and claim more than 1 million lives per year worldwide. There are multiple bacterial genes that control the pathogenesis of *Shigella*, but the *virF* gene may be the most important. This gene, located on the primary pathogenicity island of *Shigella*, encodes VirF, an AraC-family transcriptional activator that is responsible for initiating the pathogenesis cycle in *Shigella*. We have previously shown that it is possible to attenuate the virulence of *Shigella flexneri* via small molecule inhibition of VirF. In this study, we probed the mechanism of action of our small molecule inhibitors of VirF. To enable these studies, we have developed a homologous and efficient expression and purification system for VirF and have optimized two different *in vitro* VirF-DNA binding assays. We have determined that one of our HTS hit compounds inhibits VirF binding to DNA with a calculated *K*_*i*_ similar to the effective doses seen in our transcriptional activation and virulence screens. This is consistent with inhibition of DNA binding as the mechanism of action of this hit compound. We have also screened 15 commercially sourced analogs of this compound and deduced an initial SAR from the approximately 100-fold range in activities. Our four other HTS hit compounds do not inhibit DNA binding and yet they do block VirF activity. This suggests that multiple agents with different molecular mechanisms of inhibition of VirF could be developed. Pursuing hits with different mechanisms of action could be a powerful approach to enhance activity and to circumvent resistance that could develop to any one of these agents.

## Introduction

The misuse of prescription antibiotics and the overuse of antibiotics in livestock feed have greatly contributed to the rapid increase in drug-resistant bacteria in the environment. The most recent World Health Organization report on antimicrobial resistance states, “A post-antibiotic era-in which common infections and minor injuries can kill, far from being an apocalyptic fantasy, is instead a very real possibility for the 21^st^ century.” [1]. Current thinking is that one potentially effective approach to overcoming this growing problem is to target bacterial virulence rather than bacterial viability [2–5].

To validate this approach, we have begun identifying/developing an anti-virulence therapy to combat *Shigella* spp. infections. VirF is an AraC-family transcriptional activator that regulates (directly and indirectly) the transcription of all downstream virulence factors in *Shigella* spp. [6–9]. Both VirF expression and activity are tightly regulated by environmental signals (pH, temperature, osmolarity), specifically signals commonly encountered in the host cell environment [10–13]. Only under these favorable conditions can VirF directly activate the transcription of two downstream virulence genes, *virB* and *icsA* [14, 15]. VirB is a secondary transcriptional activator that is responsible for activating the transcription of other virulence genes, such as *ipaB*, *ipaC*, and, *ipaD* [12], whose gene products are involved in the construction of the Type III Secretion System and the escape from host-cell defense systems [16–18]. IcsA assembles actin polymerase on one pole of the bacterium and propels the bacterium through the infected host cells via the polymerization of host cell actin; allowing the bacterium to spread to adjacent cells [19–21]. Gene silencing studies have shown that the lack of VirB expression leads to a loss of virulence [6, 22], and that the lack of IcsA expression blocks the intra- and inter-cellular movement of *Shigella* [23, 24]. Additionally, in the infected host, *Shigella* utilizes VirF-induced IpaB to escape from macrophages [17]. These results suggest that inhibition of VirF with a small molecule should block not only the initial cellular invasion, but also prevent an active *Shigella* infection from continuing to spread from cell-to-cell and increase the efficiency of macrophage killing *Shigella*.

**Fig 1:**
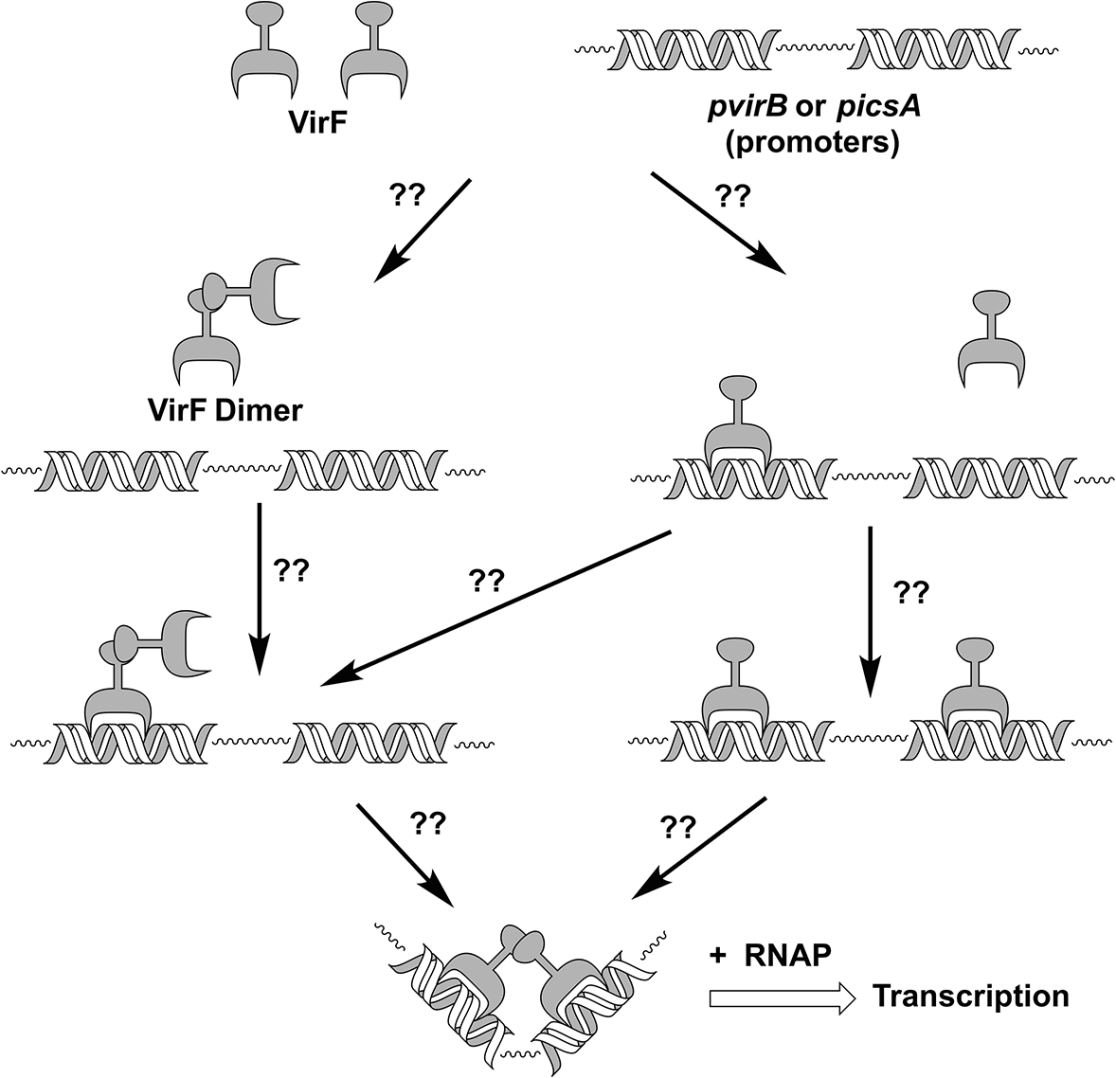
Schematic Depicting Presumptive Steps in the Activation of Transcription by VirF. By analogy to AraC and family members, it is presumed that two VirF proteins bind to two proximal DNA binding sites in a dimeric form to activate transcription. The question marks indicate that the exact steps in this process and their order (if ordered) are not yet understood.

The exact mechanism by which VirF activates transcription is not presently understood. Like AraC and most AraC family members, VirF has two domains, an N-terminal dimerization domain and C-terminal DNA binding domain. Both of these domains are necessary for *in vivo* transcriptional activation [25]. As shown in Fig 1, in order for VirF to activate transcription it must bind to the correct promoter region (either the *virB* promoter (*pvirB*) or the *icsA* promoter (*picsA*)), dimerize, and recruit RNA polymerase. The order of these events, indeed if they are ordered at all, is presently unknown. Our small molecule inhibitors could be disrupting any of these steps of the VirF gene activation process. In fact, there have been reports indicating that VirF, and/or homologous AraC-family members, can be inhibited through the blockade of DNA binding [4, 26] or self-dimerization [5]. A clearer understanding of the mechanism of action of AraC-family inhibitors would provide critical insight for furthering their development.

It has recently been shown that *S*. *flexneri* virulence can be attenuated via treatment with small molecules that inhibit VirF [26, 27]. VirF appears to be an ideal target for an anti-virulence therapy because a number of factors suggest that the likelihood of resistance to VirF inhibitors developing environmentally (outside of an infected host) should be quite low. For example, absent conditions that mimic those of an infected host, there should be little or no expression of VirF [10–13], therefore; the VirF-selective inhibitors should have no effect on *Shigella* spp. in the environment. Additionally, targeting virulence gene expression does not impair bacterial viability [28, 29], and hence, there should be little selective pressure in the environment for resistance development. Finally, in the infected host, *Shigella* utilize VirF-induced IpaB to escape from macrophages [17]. Inhibition of VirF should block this and increase the efficiency of macrophage killing *Shigella* and thereby reduce the development of resistance. Of course, these are postulates and require experimental testing to determine their validity.

**Fig 2:**
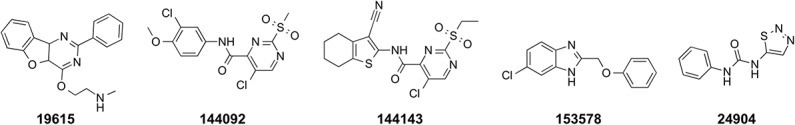
Structures of Previously Identified Small Molecule HTS Hit Compounds. The compounds are referenced by their numbers in the University of Michigan, Center for Chemical Genomics (CCG) compound libraries.

We have previously reported the identification of five promising small molecule inhibitors of VirF (see Fig 2) from a high-throughput screening campaign (over 140,000 compounds screened) and a series of follow-up assays, including tissue-culture based invasion and cell-to-cell spread assays that model aspects of the infection process [27, 30]. All five compounds inhibited VirF-driven transcriptional activation in a *Shigella*-based, β-galactosidase reporter assay with IC_50_ values in the low micromolar range (14–66 μM). Furthermore, at concentrations at or below their IC_50_s in the reporter assay, three of the compounds (**19615, 144092, 153578**) inhibited the spread of an active *S*. *flexneri* infection by approximately 75% in a tissue-culture based plaque efficiency assay, and one of the compounds (**144092**) also inhibited initial *S*. *flexneri* invasion by approximately 50% in a gentamicin protection assay. These results, supported by similar results recently published by other groups [4, 5, 26], validate our approach by providing proof of principle that small molecules can attenuate virulence; however, the mechanism by which our compounds inhibit the VirF transcriptional activation process remained unclear (see Fig 1).

In the studies herein reported, we have probed the mechanism of action of the small molecule inhibitors of VirF that we have discovered [27, 30]. To enable these studies, we have developed a homologous and efficient expression and purification system for VirF and have optimized two different *in vitro* assays which monitor VirF binding to the DNA promoter region for VirB (*pvirB*). We make the first report (to our knowledge) of a dissociation constant for VirF binding to DNA (i.e., *pvirB*) and provide strong evidence that inhibition of VirF binding to DNA is the mechanism of action of one of our hit compounds (**19615**, Fig 2). Finally, we screened a series of analogs of **19615** and have deduced an initial structure-activity relationship that we are using as a basis to further optimize our inhibitor and work towards achieving our goal of developing a novel therapy for treating shigellosis.

## Results and Discussion

To determine the mechanism of action of the compounds and further validate this approach, an efficient preparation of VirF was developed and *in vitro* assays that directly monitor VirF binding to DNA were optimized.

### MalE-VirF Purification

**Fig 3:**
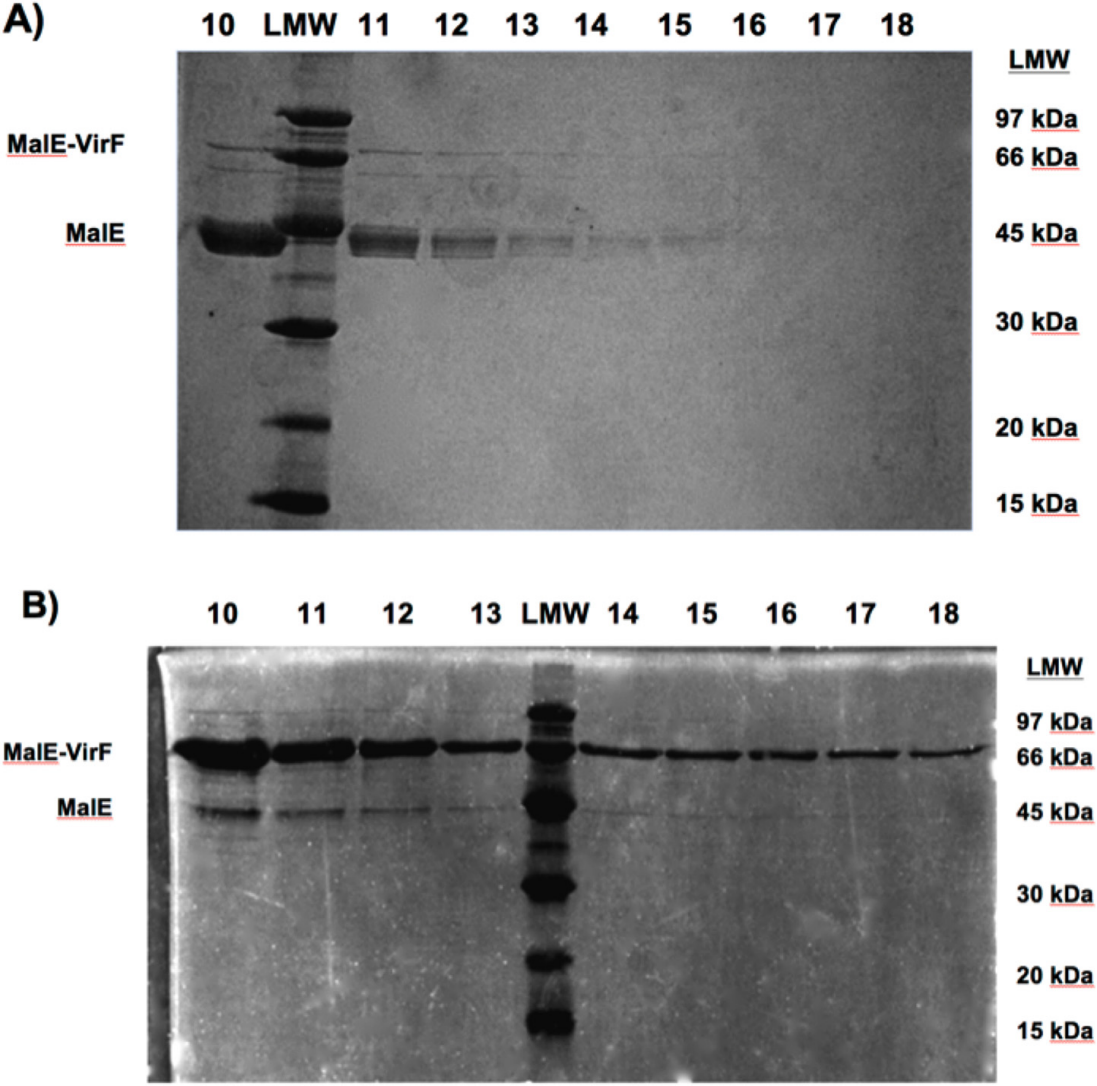
SDS-PAGE of MalE-VirF Purifications (Heterologous vs. Homologous Expression). A) SDS-PAGE analysis of MalE-VirF expression in *E*. *coli* KS1000 cells via pMALvirF expression vector. MalE-VirF is 73 kDa in weight and the main impurity, MalE is 43 kDa in weight. Numbers at the top of the gel represent the fraction (1 mL volume) number eluting off the amylose resin column. LMW stands for low molecule weight ladder; the numbers on the right-hand side of the gel correspond to the sizes of the LMW bands. B) SDS-PAGE analysis of MalE-VirF expression in *S*. *flexneri* BS103 cells via pBAD202-MALvirF expression vector. MalE-VirF is 73 kDa in weight and the main impurity, MalE, is 43 kDa in weight. Numbers at the top of the gel represent the fraction (1 mL volume) number eluting off the amylose resin column. LMW stands for low molecule weight ladder; the numbers on the right-hand side of the gel correspond to the sizes of the LMW bands.

VirF is an AraC-family transcriptional activator. AraC transcriptional activators can function monomerically or as homodimers [31]. VirF is a member of the homodimer class. Unfortunately, AraC-family members, especially those of the homodimer class, are difficult to isolate due to low solubility and poor heterologous expression [32]. Recently, Egan and co-workers reported a VirF purification protocol that utilized a previously developed maltose binding protein (MalE) fusion [14] which improves VirF solubility and allows for purification via amylose resin chromatography [26]. We had previously attempted many different protocols to purify this MalE-VirF fusion protein, but had very little success due to poor heterologous expression in various *E*. *coli* strains. The recently published protocol expressed MalE-VirF in *E*. *coli* KS1000 from an IPTG-inducible pMALvirF vector [26]. Our initial attempts to purify VirF which relied on this expression/purification system produced results typically seen in other *E*. *coli* heterologous expression systems: low overall yield and large amounts of MalE impurity (as shown via SDS-PAGE analysis in Fig 3A).

To improve the expression and purification, an arabinose-inducible vector, pBAD202-MALvirF was constructed that allowed for homologous expression of MalE-VirF in *S*. *flexneri* BS103 cells. As shown in Fig 3B, this expression system, when used with the same amylose resin purification procedure, greatly improved overall yield (approximately 1.5–2.5 mg per liter of culture) and purity of the MalE-VirF preparation (over 80% pure). Analytical gel filtration chromatography indicates that the purified MalE-VirF exists in solution as a monomer at concentrations used in subsequent experiments (see Fig A in S1 File).

### Inhibition of DNA Binding via Electrophoretic Mobility Shift Assay (EMSA)

Researchers at Paratek Pharmaceuticals recently identified a series of benzimidazole compounds [3], similar to **153578**, that inhibited the ability of LcrF, an AraC-family transcriptional activator in *Yersinia* (and subsequently a number of other AraC-family proteins), to bind to its DNA promoter region [4]. Egan and co-workers also recently found a small molecule that inhibited the ability of multiple AraC-family members, including VirF, to bind DNA [26]. These studies prompted us to focus our initial efforts on developing *in vitro* assays to monitor VirF binding to its DNA promoter site to determine if our compounds inhibit DNA binding.

**Fig 4:**
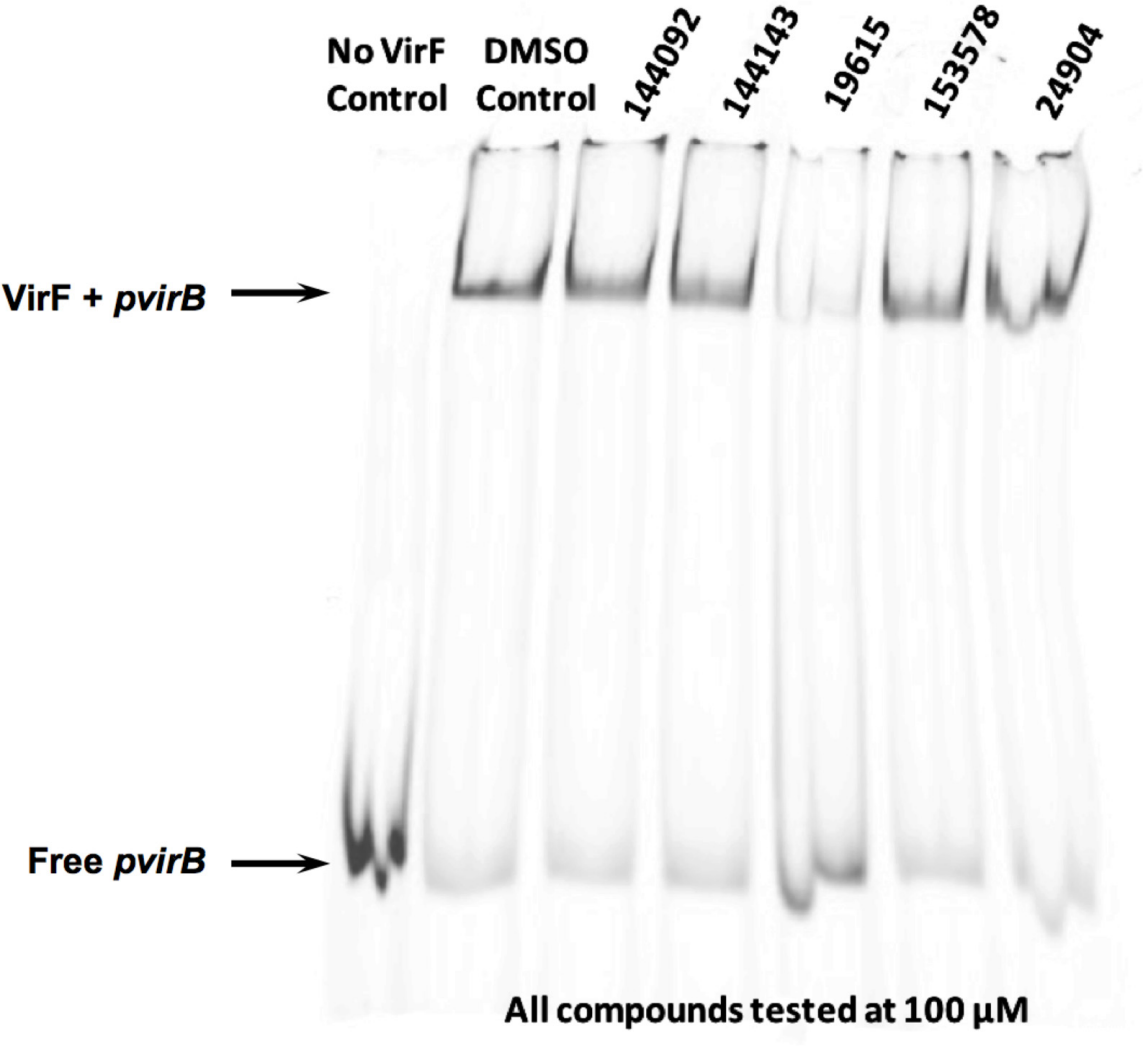
Electrophoretic Mobility Shift Assay (EMSA) PAGE of MalE-VirF Binding to *pvirB* DNA Probe in Presence of Small Molecule Hit Compounds. EMSA image shows the retardation of the 5’Cy5-*pvirB* DNA probe (0.25 μM) when incubated in the presence of MalE-VirF (1 μM). Image also shows the effect each of the five hit compounds have on MalE-VirF binding. All compounds were dosed at 100 μM, and only one compound, 19615, appeared to inhibit MalE-VirF binding to the *pvirB* DNA probe.

To monitor MalE-VirF binding to the *virB* promoter, an EMSA was optimized that utilized a fluorescently-labeled *pvirB* DNA fragment. The *pvirB* fragment was 74 bp long, with 60 bp corresponding to the previously determined [14] *pvirB* region, and 14 bp corresponding to a 5’-Cy5 labeled LUEGO (labeled universal electrophoretic gel shift oligonucleotide) site. Control experiments were conducted to verify that MalE-VirF was specifically binding to the 5’Cy5-labeled *pvirB* DNA probe (see Fig B in S1 File) and that this binding was dose-dependent. It was observed that binding of MalE-VirF to the *virB* promoter could not be monitored unless the pH of the gel/running buffer was 9.5. If the pH was lower than 9.5, the protein/DNA complex would not enter the gel matrix (data not shown). Also, the MalE tag was left on the N-terminal of VirF to help prevent VirF precipitation and aggregation during the course of the assay. The MalE-tag has been previously shown to have little effect on VirF activity [14, 27, 30] and did not prevent visualization of binding in the EMSA (see Fig 4).

After the EMSA conditions were optimized, it was used to determine if our five previously identified hit compounds (see Fig 2) could inhibit binding of MalE-VirF to the *pvirB* at 100 μM. As shown in Fig 4, one compound, **19615**, dramatically reduced binding, while the other four compounds were indistinguishable from the DMSO negative control. Surprisingly, **153578**, the benzimidazole-derivative hit compound from our HTS, had little to no effect on DNA binding under these conditions. To determine if **19615** is a totally non-specific inhibitor of protein-DNA binding, we performed a control EMSA that revealed no effect on *E*. *coli* RNA polymerase binding to the *lac* promoter in the presence and absence of **19615** (see Fig C in S1 File).

### DNA Binding via Fluorescence Polarization (FP)

**Fig 5:**
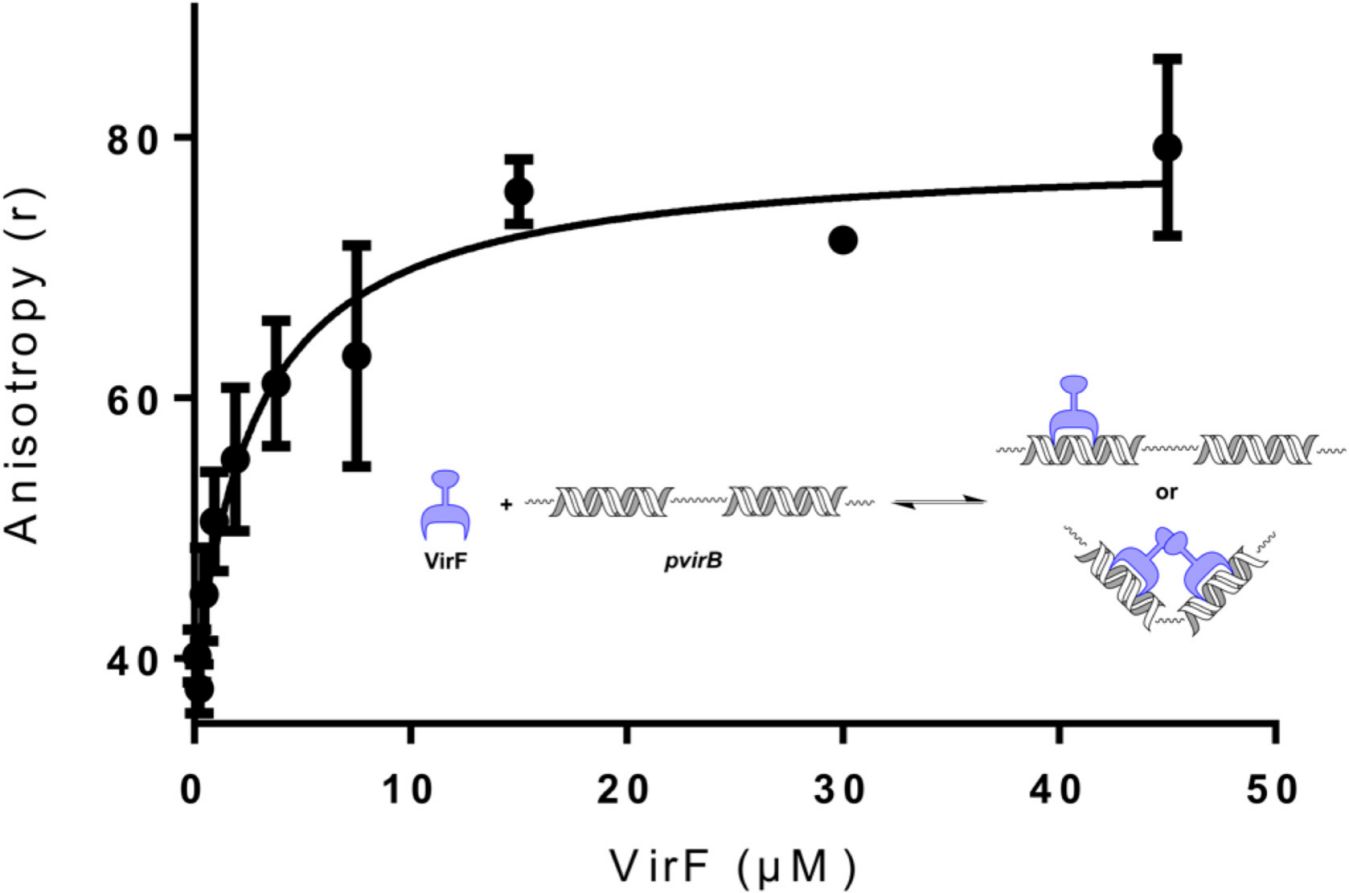
Plot of MalE-VirF Binding to the *pvirB* DNA Probe. For the assay, 5’Fluoroscein-*pvirB* DNA probe concentration was held constant at 50 nM, while MalE- VirF concentration was varied from 45 μM to 0.12 μM. The observed binding max for MalE-VirF binding was approximately r = 75, while the observed baseline (no MalE-VirF, only free *pvirB* DNA probe) was r = 42. The assay was conducted in duplicate. The inset depicts the reaction being monitored, VirF (not known if monomer or dimer) binding to the *virB* promoter.

To confirm and quantitate the results of the EMSA, a Fluorescence Polarization (FP) assay was developed. The FP assay utilized reaction conditions similar to the EMSA, with the only major change being to the *pvirB* DNA fragment. For the FP assay, the *pvirB* fragment was shortened to 60 bp with the LUEGO site being removed and was labeled with a 5’Fluoroscein instead of Cy5. These changes increased the FP signal generated in the assay by decreasing the molecular weight of the fragment and increasing the fluorescent lifetime of the probe. Control experiments were conducted to verify that: equilibrium was reached (anisotropy signal did not increase after 20 minutes), binding was specific (MalE-VirF binding to labeled *pvirB* DNA probe could be competed by unlabeled *pvirB* DNA probe and MalE-VirF would not bind to sequence scrambled DNA probe, see Fig B in S1 File), and maximum anisotropy signal was achieved. Once optimized, the FP assay was utilized to determine the *K*_*D*_ of MalE-VirF binding to the *pvirB* DNA probe. The data shown in Fig 5 was fitted with a specific binding equation (see Methods) and the experimental *K*_*D*_ was determined to be 2.8 ± 1.0 μM. To our knowledge this is the first reported *K*_*D*_ for VirF binding either of its promoter regions. It has been shown that VirF only activates the transcription of the *virB* gene when supercoiled DNA is used as a template [33]. Therefore, we should note that it is possible that our experimental *K*_*D*_, determined with a linear DNA fragment may be different than the *in vivo K*_*D*_, under physiological conditions where VirF is activating supercoiled DNA.

### Inhibition of DNA Binding via Fluorescence Polarization (FP)

**Fig 6:**
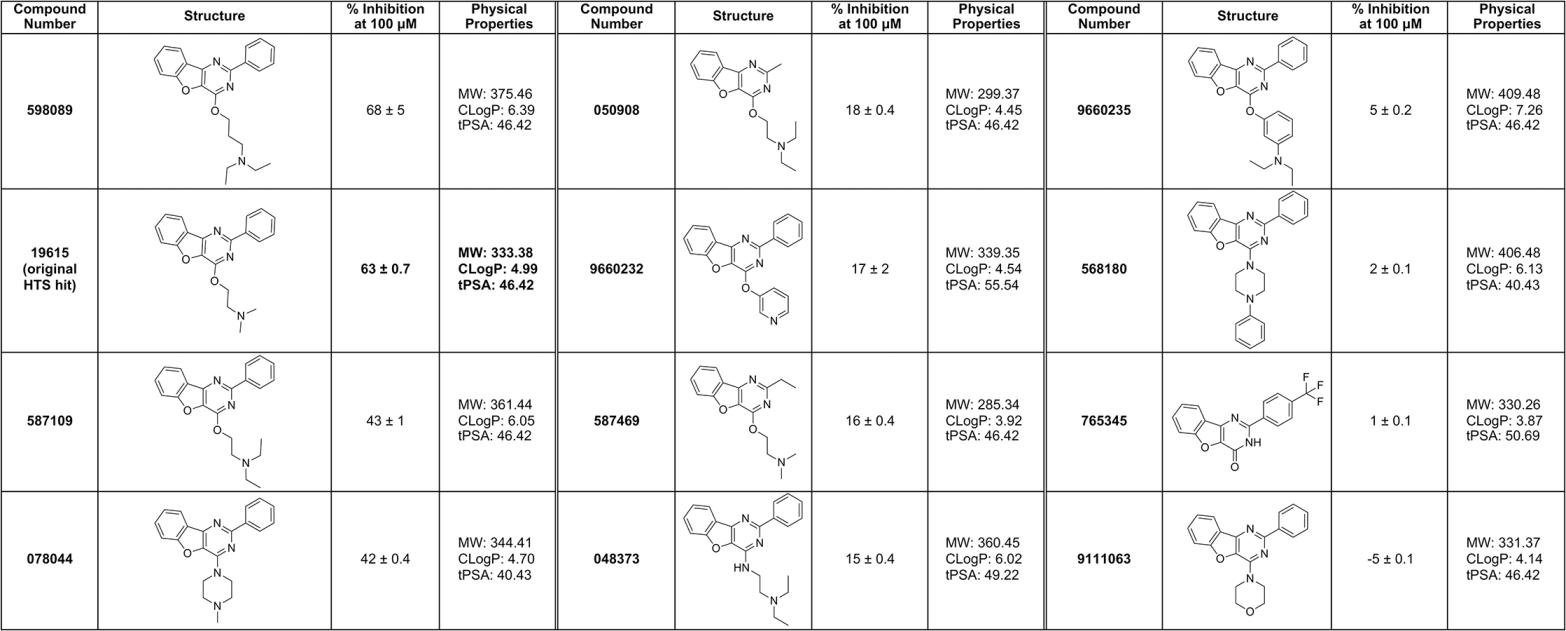
Results of 19615 Analog Testing in FP Assay.

The FP assay protocol was used to test the ability of our HTS hit compounds to inhibit MalE-VirF binding to the *pvirB* DNA probe. MalE-VirF was held constant at 20 μM, a concentration higher than its *K*_*D*_ value while still sub-saturating, to balance the magnitude of the anisotropy signal and the sensitivity to inhibition. Consistent with the EMSA study, the five compounds were tested at 100 μM, and only **19615** inhibited DNA binding (63% inhibition, see Fig 6). These results confirmed our findings in the EMSA assay, and suggest that the other four compounds are inhibiting VirF activity at different steps of the gene activation process subsequent to DNA binding. (Another, albeit unlikely possibility is that the four other compounds may be metabolized in vivo to species that do inhibit VirF-DNA binding. This will be determined as we continue to investigate their mechanisms of action.) If different molecular mechanisms of inhibition for the different compounds are confirmed, then the development of multiple compounds may circumvent any resistance and/or toxicity issues that could arise during further optimization of any one of the compounds.

**Fig 7:**
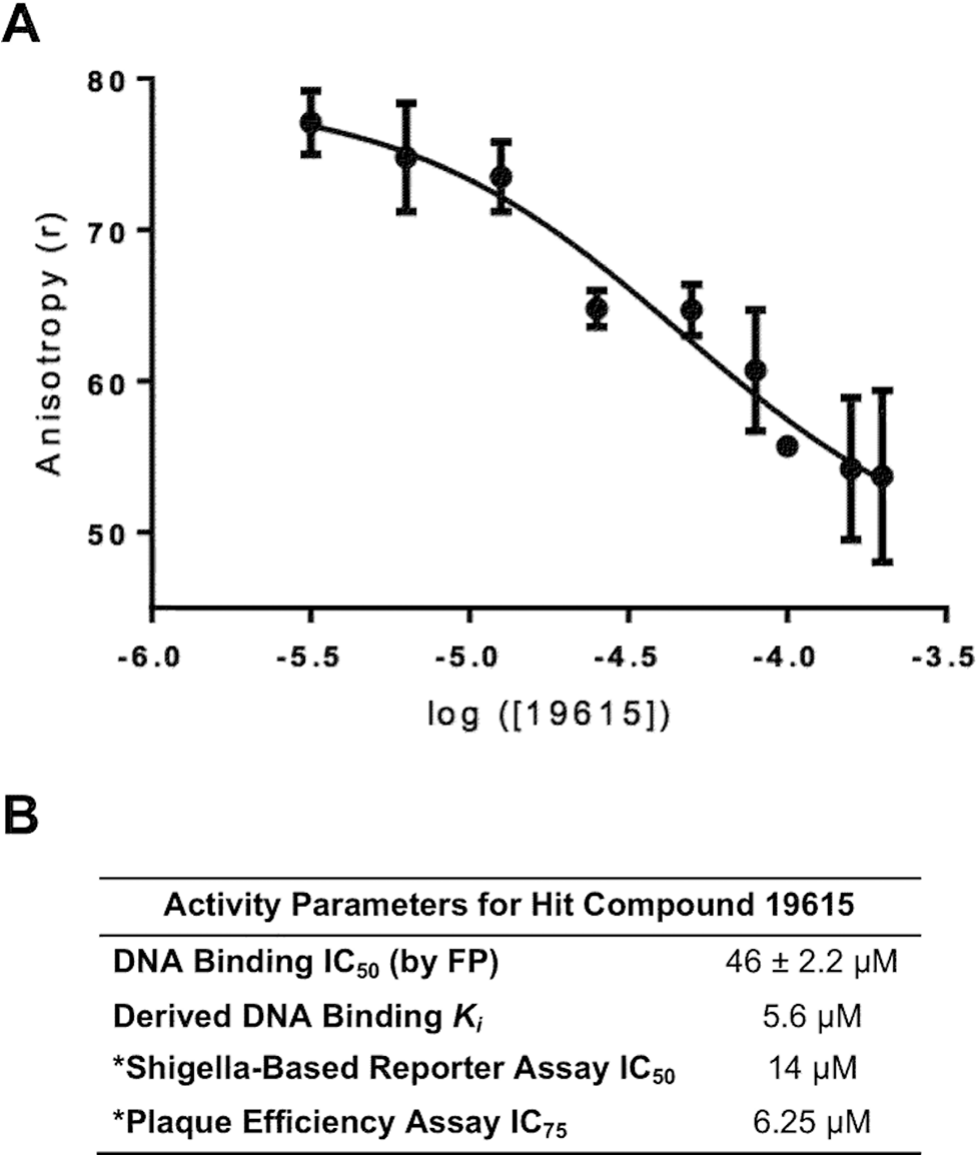
Characterization of Hit Compound 19615. A) Plot of inhibition of MalE-VirF binding to the *pvirB* DNA probe by **19615**. Assayed as in Fig 5 with the MalE-VirF concentration constant at 20 μM. B) Parameters for **19615**. An IC_50_ of 46 ± 2.2 μM for DNA binding was determined, which, by the Cheng-Prusoff equation, yields a derived *K*_*i*_ = 5.6 μM. The assay was conducted in duplicate. * Data from Emanuele et al. J. Antibiotics **67:**379–386 (2014).

To further evaluate the potency of **19615** in the FP assay, a dose-response study was performed (see Fig 7A); from which an IC_50_ of 46 ± 2.2 μM was determined. Applying this IC_50_ to the Cheng-Prusoff equation produces a *K*_*i*_ for **19615** of 5.6 μM. In our previous study [27], we determined an IC_50_ for **19615** in a *Shigella*-based, VirF-driven, β-galactosidase reporter assay to be 14 μM and showed that **19615** inhibited the spread of an active *S*. *flexneri* infection by 75% at 6.25 μM (see Fig 7B). The correlation between these results and the *K*_*i*_ for inhibition of VirF binding to DNA strongly suggest that **19615** attenuates the virulence of *S*. *flexneri* by decreasing VirF-driven transcriptional activation via inhibition of VirF-DNA binding. This, combined with the fact that **19615** was not toxic to mammalian cells or *S*. *flexneri* at the tested concentrations [27], makes **19615** an attractive candidate for further exploration.

### DNA Affinity Determination via a Fluorescence Intercalator Displacement (FID) Assay

To determine if **19615** was inhibiting VirF from binding to the *pvirB* by directly binding to the DNA a FID assay was conducted. FID assays are commonly used to establish the DNA binding affinity and selectivity of small molecules [34, 35]. In this case, the assay tested the ability of **19615** and Berenil, a known minor groove binder with a preference for AT-rich sequences [34, 36], to displace ethidium bromide from *pvirB* DNA probes. Two different *pvirB* probes were used in the study: a full length 60 bp probe, *pvirB* FID, and a 10 bp probe, *pvirB* 51–60 FID. A preliminary FID screen was conducted to determine which segment of the *pvirB* to use for the 10 bp probe and the results are shown in Table A in S1 File. As shown in Table 1, Berenil was able to displace ethidium bromide from the *pvirB* DNA probes and lower the fluorescence signal generated in the assay to produce low %fluorescence values for all experimental conditions; on the other hand, **19615** was not able to displace ethidium bromide from the *pvirB* DNA probes resulting in high %fluorescence values for all experimental conditions. These results indicate that **19615** has low affinity for the *pvirB* at concentrations used throughout the course of this study and suggest that **19615** inhibits VirF from binding to the *pvirB* via direct interaction with VirF.

**Table 1:** Results of the Fluorescence Intercalator Displacement Assay.

Compound	60 bp *pvirB* FID probe %fluorescence	10 bp *pvirB* 51–60 FID probe %fluorescence
12.5 μM **19615**	93% ± 3%	97% ± 2%
25 μM **19615**	97% ± 2%	97% ± 3%
50 μM **19615**	95% ± 2%	97% ± 2%
100 μM **19615**	93% ± 3%	95% ± 1%
12.5 μM Berenil	76% ± 2%	52% ± 1%
25 μM Berenil	67% ± 2%	51% ± 2%
50 μM Berenil	58% ± 1%	51% ± 1%
100 μM Berenil	48% ± 2%	46% ± 1%

### Initial SAR of 19615 Inhibition of VirF-DNA Binding

The next logical step towards achieving our goal of developing an anti-virulence therapy for treating shigellosis is to structurally optimize the hit compounds to improve their potency. To construct an initial SAR, a small library of analogs of compound **19615** were purchased and screened in the FP assay. The FP assay was chosen over other assays previously developed (e.g., *Shigella*-based reporter assay, tissue-culture based virulence assays) because it eliminates a number of complicating factors associated with measuring efficacy in the other assays, such as permeability and cytotoxicity. As shown in Fig 6, one of the **19615** analogs (**598089**) was essentially equal in potency to **19615** (68% versus 63% inhibition, respectively) and the rest of the compounds displayed lower %inhibition values (ranging from 43% to -5%).

**Fig 8:**
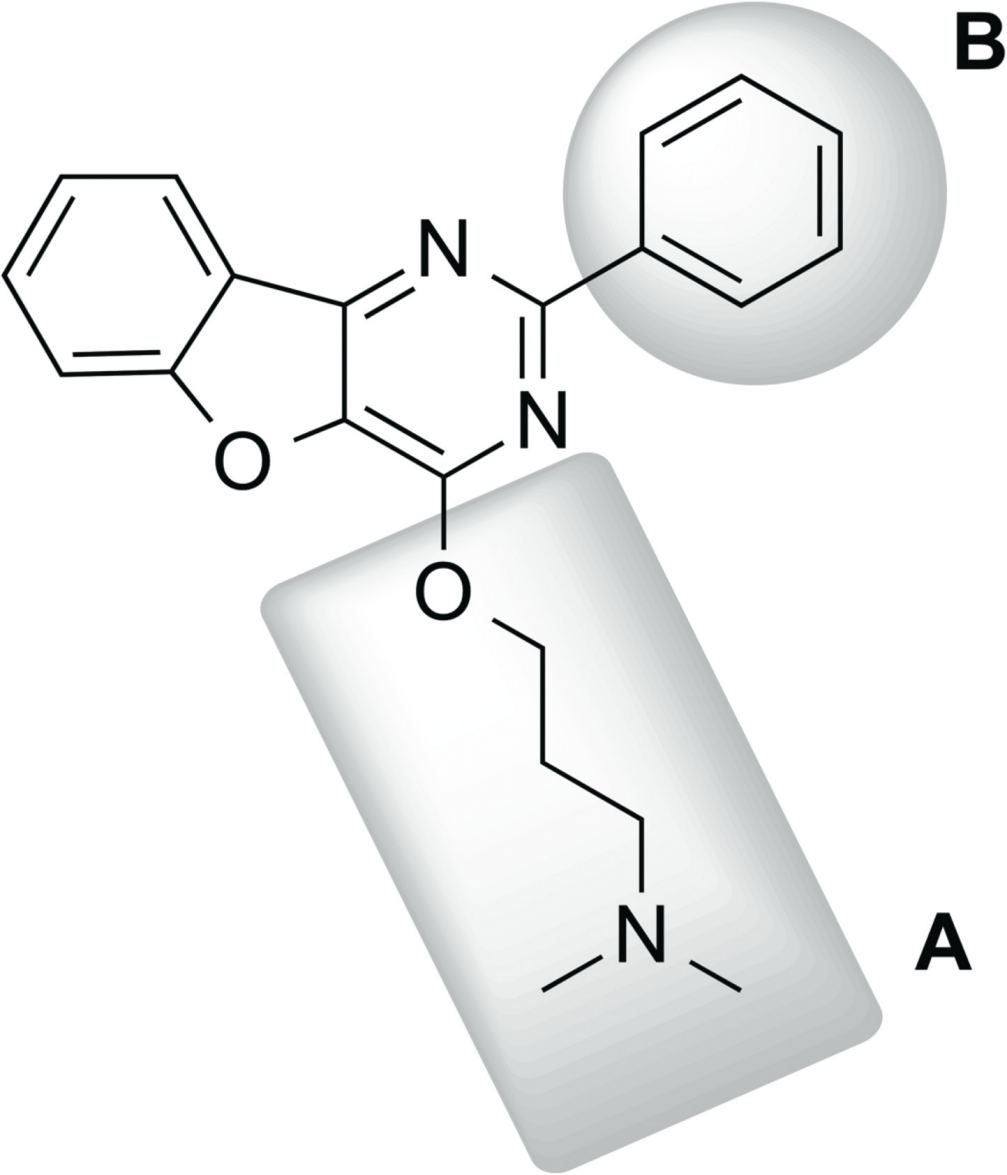
Figure Depicting Deduced SAR. Structure shown is a hybrid compound containing most promising substituents and will serve as a starting point for future synthetic SAR studies that will probe the core heterocycle. Region A: SAR data suggest the preferred substituent at this position is an ether chain with a 2–3 carbon spacer and small aminoalkyl headpiece. Region B: SAR data suggest the preferred substituent at this position is an aromatic ring, although further probing with substituted phenyl and heteroaromatic moieties is needed.

It is worth noting that the goal of testing this panel of analogs was primarily to provide supporting evidence for the mechanism of action of **19615** and secondarily to find a compound that was more potent than compound **19615**. Some notable trends identified from the data include (a) preference of a phenyl ring substituent over smaller alkyl chains attached to the pyrimidine (e.g., **19615** vs. **582610** or **587469**), (b) preference of side chain ether rather than amine linkage to pyrimidine (e.g., **587109** vs. **048373**), and (c) preference for the dimethyl amine over the diethyl amine on the ether side chain (**19615** vs. **587109** and **582610** vs. **050908**). The data also suggest that there is considerable space for favorable SAR development through the installation of additional basic side chain ethers and aromatic moieties off the pyrimidine ring. Fig 8 depicts the most promising trends that will serve as the starting point for later generations of **19615** analogs with the goal of developing a more potent inhibitor.

## Conclusion

In this report, we have determined the mechanism of VirF inhibition by compound **19615** and have constructed an initial SAR to be used for further development. In the course of the study, we made the first report of a dissociation constant for VirF binding to the *virB* promoter, and refined binding assays and a homologous expression system that will be useful in our further studies of VirF. However, at the present time, we do not yet have direct evidence to indicate that **19615**'s effects are specific to VirF versus other AraC-family transcription factors, although we have determined that **19615** does not interfere with RNAP binding to DNA (see Fig C in S1 File). There is precedent for small molecules to exhibit cross-reactivity against multiple AraC-family AraC-family members [2, 3, 37]. To examine the specificity of **19615**, we plan to test it (or a more potent analogue) in EMSAs against a variety of other AraC-family regulators that are known to activate virulence cascades in other organisms, such as ToxT from *Vibrio cholera* and LcrF from *Yersinia pestis*. Once specificity is determined, we will conduct mutagenesis studies to further probe the interactions between the compound and its target(s). We also plan on determining the mechanism of action of the other hit compounds from our high-throughput screen, conducting a synthetic SAR study of **19615** to probe the core heterocyclic structure, and continuing our work towards the development of an anti-virulence therapy to treat shigellosis.

## Material and Methods

### Reagents

All reagents were purchased from Fisher Scientific (Hampton, NH), unless otherwise specified. Compound 19615 was purchased from Vitas-M Laboratories (Moscow, Russia), while compounds **144092**, **144143**, **153578**, and **24904** were purchased from ChemDiv (San Diego, CA). Analogs of **19615** were purchased through Aldrich Market Select (St. Louis, MO). Berenil was purchased from Sigma Aldrich (St. Louis, MO). Vendors for other reagents are specified throughout the text.

### Strains and Plasmids

*Escherichia coli* KS1000 was purchased from New England Biolabs (Ipswich, MA). A virulence plasmid-cured derivative of wild-type *S*. *flexneri*, BS103 [38], was also used in this study. Two different expression vectors were used in this study: pMALvirF and pBAD202-MALvirF. Both vectors encode for a maltose binding protein–VirF fusion protein, MalE-VirF. pMALvirF was constructed by cloning the *virF* gene into the vector, pMAL-c2x (New England Biolabs), as previously described [14, 30]. pBAD202-MALvirF was constructed using the pBAD directional TOPO expression kit (Invitrogen, Carlsbad, CA). Briefly, the *malE-virF* fusion gene was amplified from pMALvirF via polymerase chain reaction to include a 5’-*Nco*I restriction site before the start codon of *malE-virF*. The amplified gene was then subcloned into pBAD202 via a directional TOPO® cloning reaction. The resulting vector was then subjected to *Nco*I restriction digestion (5 units, 1 hour, 37°C) to remove the N-terminal His-Patch thioredoxin leader sequence from pBAD202, and form pBAD202-MALvirF. The sequences of both expression vectors were confirmed by DNA sequencing (DNA Sequencing Core Facility, University of Michigan).

### Expression and Purification of MalE-VirF

Initial expression and purification experiments were conducted using pMALvirF and *E*. *coli* KS1000 as previously described [26]. Subsequent experiments utilized pBAD202-MALvirF to express MalE-VirF in *S*. *flexneri* BS103 as follows. Using a MicroPulser Electroporator (BioRad, Hercules, CA), pBAD202-MALvirF was transformed into electrocompetent *S*. *flexneri* BS103 cells. Starter cultures (10 mL) of pBAD202-MalVirF BS103 were grown overnight in 2xTY broth (16 g bactotryptone, 10 g yeast extract, 5 g NaCl per liter of water) supplemented with kanamycin (50 μg/mL) at 37°C with shaking. The following day the starter culture was used to inoculate 1 L of 2xTY broth supplemented with kanamycin. The cells were grown to an OD_600_ = 0.5. Expression of MalE-VirF was induced with the addition of arabinose (0.2% final concentration) and the culture continued to shake at 37°C for an additional 5 hours. Cells were then harvested via centrifugation (6000 X g, 4°C, 15 minutes) and were stored overnight at -20°C. The next day the cells were resuspended in 20 mL of amylose resin binding buffer (20 mM Tris-HCl, 500 mM NaCl, 1 mM EDTA, 1 mM, pH = 7.4) supplemented with phenylmethylsulfonyl fluoride (0.1 mM) and 20 μL of lysonase bioprocessing reagent (EMD Millipore, Billerica, MA). Cells were slowly stirred for 10 minutes at room temperature and were then immediately placed on ice and kept on ice or at 4°C for the remainder of the procedure. Cells were lysed via sonication (8 cycles, 10 second pulse time, 2 minute intervals, max pulse setting) utilizing a ultrasonic XL2020 sonicator (Misonix, Farmingdale, NY). Following sonication, cellular debris were removed via centrifugation (25,000 X g, 4°C, 40 minutes). The resultant supernatant was then applied to a 10 mL column of amylose resin (New England Biolabs) by gravity flow. Before addition of the supernatant the column was washed with 8 column volumes of amylose resin binding buffer. Following addition of the supernatant, the column was washed with 12 column volumes of amylose resin binding buffer. MalE-VirF was eluted from the column in 1 mL fractions of amylose resin elution buffer (amylose resin bind buffer plus 15% glycerol (wt/vol) and 10 mM maltose). Fractions were analyzed by SDS-PAGE. Fractions containing MalE-VirF were pooled, concentrated to approximately 6.5 mg/mL using Amicon Ultra-15 centrifugal units (EMD Millipore), and stored in liquid nitrogen for future use.

### Analytical Gel Filtration

Analytical gel filtration chromatography was used to determine the oligomeric state of purified MalE-VirF. Briefly, MalE-VirF (0.75 mg/mL) was applied to a Superose 12 column, which was equilibrated with amylose resin binding buffer using an AKTA FPLC system (both from GE Healthcare Life Sciences, Piscataway, NJ). The sample was run through the column at a flow rate of 0.75 mL/min using amylose resin binding buffer. Eluted proteins were detected spectrophotometrically at 280 nm. The oligomeric state of MalE-VirF was determined by comparison to a previously generated 4-point molecular weight calibration curve specific to the Superose 12 column.

### DNA Probe Hybridization

DNA probes were utilized in both EMSA, FP, and FID assays. The sequences of the oligonucleotides were based on previous studies [26, 39] and are listed in Table 2. All oligonucleotides were purchased from Invitrogen, except LUEGO which was purchased from Integrated DNA Technologies (Coralville, IA). Each oligonucleotide was brought up to a final concentration of 10 μM in TE/NaCl buffer (10 mM Tris-HCl, 1 mM EDTA, 50 mM NaCl, pH = 8.0). For EMSA experiments, oligonucleotides were mixed at the following ratio: 10 volumes LUEGO, 5 volumes Top, 1 volume Bottom. For FP and FID experiments, oligonucleotides were mixed 1:1 (Top:Bottom). Annealing was performed using a Mastercycler Nexus thermocycler (Eppendorf, Hauppauge, NY) with the following program: 94°C for 2 minutes, cool down at 2°C/sec to 70°C and hold for 2 minutes, cool down at 0.1°C/sec to 20°C and hold for 2 minutes.

**Table 2:** Oligonucleotides used in EMSA, FP, and FID experiments.

Name	Sequence (5’-3’)	Modification	Length (bases)
*pvirB* Top EMSA	AGAATATTATTCTTTTATCCAATAAAGATAAATTGCATCAATCCAGCTATTAAAATAGTA	None	60
*pvirB* Bottom EMSA	TACTATTTTAATAGCTGGATTGATGCAATTTATCTTTATTGGATAAAAGAATAATATTCTCCAGACCAGGGCAC	None	74
*pScram* Top EMSA	TAAGTCCTAAATGGAAATTAAATTACGTAATTCACAAATATAGTATGATCATTTATATCA	None	60
*pScram* Bottom EMSA	TGATATAAATGATCATACTATATTTGTGAATTACGTAATTTAATTTCCATTTAGGACTTACCAGACCAGGGCAC	None	74
LUEGO	GTGCCCTGGTCTGG	5’-Cy5	14
*pvirB* Top FP	AGAATATTATTCTTTTATCCAATAAAGATAAATTGCATCAATCCAGCTATTAAAATAGTA	5’-Fluoroscein	60
*pvirB* Bottom FP	TACTATTTTAATAGCTGGATTGATGCAATTTATCTTTATTGGATAAAAGAATAATATTCT	None	60
*pScram* Top FP	TAAGTCCTAAATGGAAATTAAATTACGTAATTCACAAATATAGTATGATCATTTATATCA	5’-Fluoroscein	60
*pScram* Bottom FP	TGATATAAATGATCATACTATATTTGTGAATTACGTAATTTAATTTCCATTTAGGACTTA	None	60
*pvirB* Top FID	AGAATATTATTCTTTTATCCAATAAAGATAAATTGCATCAATCCAGCTATTAAAATAGTA	None	60
*pvirB* Bottom FID	TACTATTTTAATAGCTGGATTGATGCAATTTATCTTTATTGGATAAAAGAATAATATTCT	None	60
*pvirB* (51–60) Top FID	TAAAATAGTA	None	10
*pvirB* (51–60) Bottom FID	TACTATTTTA	None	10

### DNA Binding via Electrophoretic Mobility Shift Assay (EMSA)

Reactions for the EMSAs were incubated in a 37°C water bath for 15 minutes. Reactions (15 μL total volume) were composed of 6 μL *pvirB* EMSA DNA probe (0.25 μM), 6 μL of either MalE-VirF (1.0 μM) or native gel loading buffer (Tris-HCl 0.3 M, 50% glycerol, 0.05% bromophenol blue, pH 7.0), 1 μL of salmon sperm DNA (0.7 mg/mL, Invitrogen), 0.5 μL BSA (0.07 mg/mL), and 1.5 μL of either DMSO or compound (100 μM). A 6% native polyacrylamide gel (29:1 acrylamide to bis-acrylamide ratio) was made with 0.25X TBE buffer (22 mM Tris Base, 22 mM boric acid, 0.5 mM EDTA, pH 9.5) for the EMSA. The gel was electrophoresed for 1 hour at 150 V in 0.25X TBE buffer at 4°C before samples were loaded. After the reaction solutions (12 μL) were loaded onto the gel, the gel was electrophoresed for an additional hour at 150 V and 4°C. The gel was then visualized using FluorChem M gel imager (Protein Simple, Santa Clara, CA) with a 607 nm excitation wavelength and a 710 nm emission filter.

### DNA Binding via Fluorescence Polarization (FP)

The FP assays were conducted in duplicate in black, half-area, 96-well plates (Corning, Twerksbury, MA). First, 30 μL of 5’-Fluoroscein-*pvirB* DNA probe working standard was added to appropriate wells of the microplate. The *pvirB* DNA working standard was in TE/NaCl buffer (see above) supplemented with BSA (0.07 mg/mL) and salmon sperm DNA (0.7 mg/mL). Next, 30 μL of varying concentrations of MalE-VirF in amylose resin elution buffer (see above) was added to appropriate wells of the microplate. The final concentration of *pvirB* DNA was either 50 nM (for test wells) or none (for blank wells) and the final concentrations of MalE-VirF ranged from 45 μM to 0.12 μM (for test wells) or none (for control wells). For each concentration of MalE-VirF tested, a blank well was also set up that included all reagents except *pvirB* DNA. The microplate was then incubated for 20 minutes at 37°C and anisotropy was determined using a SpectraMax M5 plate reader (Molecular Devices, Sunnyvale, CA). Data was acquired using an excitation wavelength equal to 490 nm and an emission wavelength equal to 520 nm. To determine anisotropy the following equation was used:

$$\mathrm{Anisotropy}=\frac{F_{∥}-G^{*}F_{⊥}}{F_{∥}+2^{*}G^{*}F_{⊥}}{}^{*}1000$$

**w**here: F_**ǁ**_ = fluorescence intensity parallel to excitation source after blanking, F_**┴**_ = fluorescence intensity perpendicular to excitation source after blanking, and G = G-factor (correction for polarization bias of the detection system). After anisotropy was determined, data were plotted using GraphPad Prism (La Jolla, Ca) and fit to the following equation:

$$Y=B_{\mathrm{MAX}}{}^{*}\;\left(\frac{X}{K_{D}}{}^{*}X\right)+b$$

where Y = specific binding, K_D_ = dissociation constant, B_MAX_ = maximum binding, X = MalE-VirF concentration, and b = y-intercept.

On average, there was a 65% increase in anisotropy when VirF was added to the DNA. Within a single set of experiments, the DNA (alone) fluorescence anisotropy values varied ~4%., whereas, between experiments these anisotropy values varied ~15%. The latter variation is most likely due to differences in the efficiency of duplex DNA formation.

### Inhibition of DNA Binding via Fluorescence Polarization (FP), IC_50_ Determination

The FP protocol listed above was adapted to determine the IC_50_ of the small molecule hit compounds. Compounds (ranging from 200 μM to 3.1 μM) or DMSO for controls (1% final concentration) were added to the *pvirB* DNA probe working standard prior to addition to the microplates. For IC_50_ determination, the MalE-VirF and *pvirB* DNA probe concentrations were held constant at 20 μM and 50 nM, respectively. Positive controls were established to determine 100% VirF inhibition (no MalE-VirF present, 50 nM *pvirB* DNA probe) and negative controls were set up to determine no VirF inhibition (1% DMSO, 20 μM MalE-VirF, and 50 nM *pvirB* DNA probe). Once again blanks were set up to include all reagents, except *pvirB* DNA probe. All reactions were run in duplicate. Anisotropy was calculated as listed above and data were plotted using GraphPad Prism then fit to the following equation:

$$Y=\mathrm{Bottom}+(\mathrm{Top}-\mathrm{Bottom})/(1+10^{\left({\mathrm{X-logIC}}_{50}\right)})$$

where: Y = anisotropy, X = log of compound concentration (M), Top and Bottom = plateaus in anisotropy units, and LogIC_50_ = log of the concentration that inhibits 50% of VirF binding. In the presence of the compounds (no VirF), a small increase (~6%) in the DNA anisotropy was observed. This did not change over the concentrations tested and may be due to electrostatic interactions between the compounds and the DNA (assuming that the lowest compound concentration of ~3 μM would saturate the 50nM DNA probe).

### Inhibition of DNA Binding via FP, %Inhibition Determination

The FP protocol used for IC_50_ determination was adapted to test a library of small molecule inhibitors of VirF. All steps were identical to the above protocol except compounds were tested at only 100 μM final concentration and positive control samples included 100 μM of compound. Once again blanks were set up to include all reagents, except *pvirB* DNA probe, and all reactions were run in duplicate. Anisotropy was calculated as listed above, and %inhibition was calculated as follows:

$$\%\mathrm{inhibition}=\frac{r_{\mathrm{neg}}-r_{\mathrm{test}}}{r_{\mathrm{neg}}}{}^{*}100$$

where: r_neg_ = average anisotropy for negative controls and r_test_ = average anisotropy in presence of compound. Note: All r_neg_ and r_test_ values were normalized to positive control values.

### Fluorescence Intercalator Displacement (FID) Assay

The FID protocol was based on previously published studies [34, 40] and was conducted in triplicate. For the assay, black, half-area, 96-well plates (Corning, Twerksbury, MA) were used. First, 70 μL of ethidium bromide in Tris Buffer (0.1 M Tris HCl, 0.1 M NaCl, pH 8.0) was added to all wells. The concentration of ethidium bromide was contingent upon the length of the DNA probe that was used. For the 60 bp *pvirB* FID DNA probe, the final ethidium bromide concentration used was 45 μM; whereas, for the 10 bp *pvirB* (51–60) FID DNA probe, the final ethidium bromide concentration used was 7.5 μM. Next, 10 μL of either *pvirB* FID DNA probes (10 or 60 bp) in TE/NaCl buffer (see above) or 10 μL of TE/NaCl buffer (background fluorescence control) were added to the appropriate wells of the plate. The final concentrations of the *pvirB* FID DNA probes were 1.5 μM. Lastly, 20 μL of test compound (either **19615** or Berenil) in 10% DMSO Tris Buffer or 20 μL of 10% DMSO Tris Buffer (100% fluorescence control) were added to the appropriate wells of the plate. The final concentrations of each test compound ranged from 12.5 μM to 100 μM. After the addition of all reagents, the plates were protected from light and allowed to equilibrate for 30 min on an orbital shaker at room temperature. Following equilibration, fluorescence was measured (545 nm emission, 595 nm excitation) using a Biotek Synergy H1 plate reader (Winooski, VT). The average background fluorescence was subtracted from all data generated and %fluorescence was calculated for all wells containing compound via the following formula:

$$\%\;\mathrm{fluorescence}=\frac{F_{\mathrm{test}}}{F_{100\%}}{}^{*}100$$

where: F_test_ = the average fluorescence value obtained for each test compound and F_100%_ = the average fluorescence value obtained for each 100% fluorescence control.

## Supporting Information

S1 File Fig A: Analytical Gel Filtration Results. I) Chromatogram depicting elution of MalE-VirF (11.04 mL) from Superose 12 column. II) Chromatogram and four-point calibration curve for Superose 12 column used to determine molecular weight of MalE-VirF in S1-A Fig. **Fig B: Negative Controls for EMSA and FP assay.** I) EMSA image shows the retardation of the 5’Cy5-*pvirB* DNA probe (0.25 μM) when incubated in the presence of MalE-VirF (1 μM) and shows no retardation of the 5’Cy5-*pScram* DNA probe (0.25 μM) when incubated in the presence of MalE-VirF (1 μM). II) Graph depicting the anisotropy values generated in the FP assay for the 5’Fluorescein *pScram* probe alone (r = 39) and in the presence of MalE-VirF (r = 36). Experiments were conducted in duplicate with 50 nM *pScram* and 20 μM MalE-VirF. **Table A: Fluorescence Intercalator Displacement Assay with 10 bp *pvirB* Probes.** *pvirB 51–60 was selected for use in the dose-response FID assay since it was sensitive to Berenil (67%) and was the most sensitive to 19615 (92%) in this study. The differential affinity of Berenil for the various 10 BP fragments reflects its preference for specific AT-rich sequences. **Fig C: EMSA depicting *E. coli* RNA Polymerase (RNAP) Binding to the *lac* Promoter (*plac*) in the Presence of 19615.** EMSA image shows the retardation of a 5’Cy5-*plac* DNA probe (0.25 μM) when incubated in the presence of *E. coli* RNAP (2.7 μM) and also shows that compound 19615 has no effect on RNAP binding when tested at 100 μM. For the EMSA a hybrid 2% acrylamide, 1% agarose gel was used which was made with and ran in a 1X TGE buffer (25 mM Tris base, 190 mM glycine, 1 mM EDTA, pH 8.3). The sequence of the 5’Cy5-*plac* DNA probe is as follows: 5’-gtgccctggtctggTTAGGCACCCCAGGCTTTACACTTTATGCTTCCGGCTCGTATAATGTGTGGAATTGTGAG-3’ (lowercase text represents LUEGO sequence, uppercase text represents *lac* promoter sequence).
(DOCX)
