# Supplementary material for: Mechanism of Action and Initial, In Vitro SAR of an Inhibitor of the Shigella flexneri Virulence Regulator VirF
Source: PLoS One. 2015 Sep 9;10(9):e0137410. doi: 10.1371/journal.pone.0137410 (PMC4564171; doi:10.1371/journal.pone.0137410)
Supplement: S1 File — I) Chromatogram depicting elution of MalE-VirF (11.04 mL) from Superose 12 column. II) Chromatogram and four-point calibration curve for Superose 12 column used to determine molecular weight of MalE-VirF in S1-A Fig. Fig B: Negative Controls for EMSA and FP assay. I) EMSA image shows the retardation of the 5’Cy5-pvirB DNA probe (0.25 μM) when incubated in the presence of MalE-VirF (1 μM) and shows no retardation of the 5’Cy5-pScram DNA probe (0.25 μM) when incubated in the presence of MalE-VirF (1 μM). II) Graph depicting the anisotropy values generated in the FP assay for the 5’Fluorescein pScram probe alone (r = 39) and in the presence of MalE-VirF (r = 36). Experiments were conducted in duplicate with 50 nM pScram and 20 μM MalE-VirF. Table A: Fluorescence Intercalator Displacement Assay with 10 bp pvirB Probes. *pvirB 51–60 was selected for use in the dose-response FID assay since it was sensitive to Berenil (67%) and was the most sensitive to 19615 (92%) in this study. The differential affinity of Berenil for the various 10 BP fragments reflects its preference for specific AT-rich sequences. Fig C: EMSA depicting E. coli RNA Polymerase (RNAP) Binding to the lac Promoter (plac) in the Presence of 19615. EMSA image shows the retardation of a 5’Cy5-plac DNA probe (0.25 μM) when incubated in the presence of E. coli RNAP (2.7 μM) and also shows that compound 19615 has no effect on RNAP binding when tested at 100 μM. For the EMSA a hybrid 2% acrylamide, 1% agarose gel was used which was made with and ran in a 1X TGE buffer (25 mM Tris base, 190 mM glycine, 1 mM EDTA, pH 8.3). The sequence of the 5’Cy5-plac DNA probe is as follows: 5’-gtgccctggtctggTTAGGCACCCCAGGCTTTACACTTTATGCTTCCGGCTCGTATAATGTGTGGAATTGTGAG-3’ (lowercase text represents LUEGO sequence, uppercase text represents lac promoter sequence). (DOCX) [file pone.0137410.s001.docx]

**Figure A: Analytical Gel Filtration Results. *Anthony A. Emanuele and George A. Garcia***

**
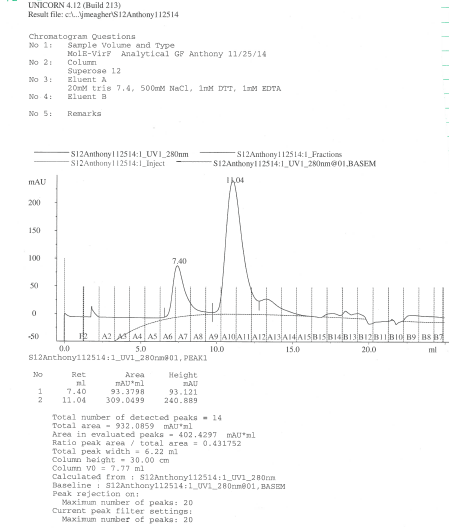

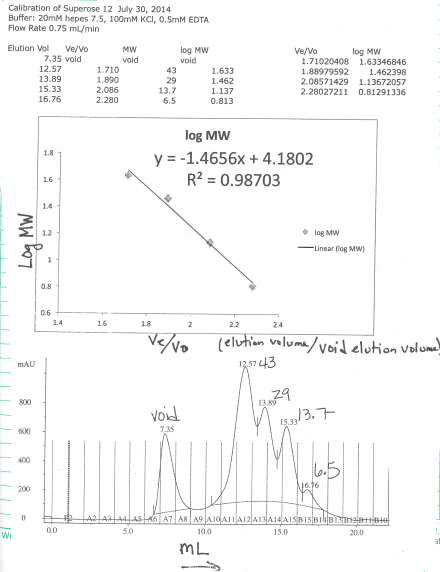
I II**

I) Chromatogram depicting elution of MalE-VirF (11.04 mL) from Superose 12 column. II) Chromatogram and four-point calibration curve for Superose 12 column used to determine molecular weight of MalE-VirF in I.

**Figure B: Negative Controls for EMSA and FP assay.**

**I**

**II**

I) EMSA image shows the retardation of the 5’Cy5-*pvirB* DNA probe (0.25 μM) when incubated in the presence of MalE-VirF (1 μM) and shows no retardation of the 5’Cy5-*pScram* DNA probe (0.25 μM) when incubated in the presence of MalE-VirF (1 μM). II) Graph depicting the anisotropy values generated in the FP assay for the 5’Fluorescein *pScram* probe alone (r = 39) and in the presence of MalE-VirF (r = 36). Experiments were conducted in duplicate with 50 nM *pScram* and 20 μM MalE-VirF.

**Table A: Fluorescence Intercalator Displacement Assay with 10 bp *pvirB* Probes.**

| **10 bp *pvirB* Fragment** | **19615 (2 μM) %fluorescence** | **Berenil (2 μM) %fluorescence** |
| --- | --- | --- |
| *pvirB* 1-10 (5’-AGAATATTAT-3’) | 93% ± 6% | 45% ± 2% |
| *pvirB* 11-20 (5’-TCTTTTATCC -3’) | 95% ± 3% | 66% ± 0% |
| *pvirB 2*1-30 (5’-AATAAAGATA -3’) | 92% ± 1% | 81% ± 4% |
| *pvirB 3*1-40 (5’-AATTGCATCA -3’) | 94% ± 2% | 89% ± 5% |
| *pvirB 4*1-50 (5’-ATCCAGCTAT -3’) | 94% ± 1% | 96% ± 2% |
| *pvirB 5*1-60 (5’-TAAAATAGTA -3’)* | 92% ± 0% | 67% ± 0% |

**pvirB* 51-60 was selected for use in the dose-response FID assay since it was sensitive to Berenil (67%) and was the most sensitive to 19615 (92%) in this study. The differential affinity of Berenil for the various 10 BP fragments reflects its preference for specific AT-rich sequences.

**Figure C: EMSA depicting *E. coli* RNA Polymerase (RNAP) Binding to the *lac* Promoter (*plac*) in the Presence of 19615.** EMSA image shows the retardation of a 5’Cy5-*plac* DNA probe (0.25 μM) when incubated in the presence of *E. coli* RNAP (core and holoenzyme with sigma70 at 2.7 μM) and also shows that compound 19615 has no effect on RNAP binding when tested at 100 μM. For the EMSA a hybrid 2% acrylamide, 1% agarose gel was used which was made with and ran in a 1X TGE buffer (25 mM Tris base, 190 mM glycine, 1 mM EDTA, pH 8.3). The sequence of the 5’Cy5-*plac* DNA probe is as follows: 5’-gtgccctggtctggTTAGGCACCCCAGGCTTTACACTTTATGCTTCCGGCTCGTATAATGTGTGGAATTGTGAG-3’ (lowercase text represents LUEGO sequence, uppercase text represents *lac* promoter sequence).
